# Supplementary material for: The acute effect of metabolic cofactor supplementation: a potential therapeutic strategy against non‐alcoholic fatty liver disease
Source: Mol Syst Biol. 2020 Apr 27;16(4):e9495. doi: 10.15252/msb.209495 (PMC7184219; doi:10.15252/msb.209495)
Supplement: Supplementary file 1 — Appendix [file MSB-16-e9495-s002.pdf]

Table of Contents

The acute effect of metabolic cofactor supplementation, a potential therapeutic strategy against non-alcoholic fatty liver disease

Cheng Zhang[1,2,#], Elias Bjornson[3,4,#], Muhammad Arif[1,#], Abdellah Tebani[1], Alen Lovric[1,§], Rui Benfeitas[1,¤], Mehmet Ozcan[1], Kajetan Juszczak[1], Woonghee Kim[1], Jung Tae Kim[1], Gholamreza Bidkhori[5], Marcus Ståhlman[3], Per-Olof Bergh[3], Martin Adiels[3], Hasan Turkez[6], Marja-Riitta Taskinen[7], Jim Bosley[8], Hanns-Ulrich Marschall[3], Jens Nielsen[4], Mathias Uhlén[1], Jan Borén[3,*], Adil Mardinoglu[1,5,*]

Appendix Table S1
Appendix Table S2
Appendix Table S3

Appendix Figure S1
Appendix Figure S2
Appendix Figure S3
Appendix Figure S4
Appendix Figure S5
Appendix Figure S6

Appendix Table S1. Observations and measurements

SIG-41				
**Group 1 females, Dos level 304 mg/kg**				
**Observation**	**Number of animals**	**Number of obs./day**	**From study day**	**To study day**
Nothing abnormally detected (NAD)	3	21	1	7
PILO	2	2	1	1
Excessive chewing (EXCH)	2	2	1	1

SIG-41				
**Group 2 females, Dos level 3040 mg/kg**				
**Observation**	**Number of animals**	**Number of obs./day**	**From study day**	**To study day**
Nothing abnormally detected (NAD)	3	21	1	7
Plouing in the bedding (PLOU)	2	11	2	7
Pilo erecction (PILO	3	3	1	1
Excessive chewing (EXCH)	3	3	1	1
Motor activity decreased (MADE)	2	2	1	1
Eyes half shut (EYHS)	3	3	1	1

SIG-41				
**Group 3 females, Dos level 11820/7880 mg/kg**				
**Observation**	**Number of animals**	**Number of obs./day**	**From study day**	**To study day**
Nothing abnormally detected (NAD)	3	21	1	7
Ploughing in the bedding (PLOU)	3	18	1	7
Pilo erection (PILO)	3	3	1	1
Excessive chewing (EXCH)	3	3	1	1
Vocalisation during handling (VODH)	1	2	6	7
Difficulty dosing (DIDO)	1	1	3	3
Ataxia (ATAX)	2	2	2	2
Cyanosis (CYAN)	1	1	2	2
Respiration irregular (REIR)	1	2	1	2
Respiration noisy (RENO)	1	1	1	1
Motor activity decreased (MADE)	3	5	1	2
Eyes half shut (EYHS)	3	4	1	2

Observations:	Observations were made daily after dosing until "No Adverse Finding" is observed and recorded. If adverse findings were observed, they were recorded during 0-30 MAD, 1-6 HAD, and 6-24 HAD, or more frequently if found necessary.
Body weight	Body weights were recorded prior to first dosing Day 1 and anytime if decline in general condition was observed.

Appendix Table S2. Haematology data

SIG-41				WBC	LYM	MONO	GRAN	LYM	MONO	GRAN	HGB	HCT	RBC	MCV	MCH	MCHC	RDW%	RDWa	PLT	MPV
ID/Animal no	Gender	Group	/Analysis date	(10^9/L)	(10^9/L)	(10^9/L)	(10^9/L)	%	%	%	g/L	%	(10^{12}/L)	fL	pg	g/dL	%	fl	(10^9 /L)	fL
1	F	1	20-Dec-17	8.1	7.2	0.2	0.7	88.9	1.4	9.7	152	42.5	8.35	50.9	18.2	357	17.9	32.9	574	5.8
2	F	1	20-Dec-17	5.4	4.7	0.1	0.6	86.5	2.1	11.4	145	39.4	7.93	49.7	18.3	369	18.6	32.8	632	5.8
3	F	1	20-Dec-17	6.4	4.9	0.2	1.3	77.2	2.5	20.3	151	39.8	8.02	49.6	18.8	380	18.1	32.3	592	6.0
4	F	2	20-Dec-17	7.7	6.0	0.4	1.3	78.3	4.9	16.8	152	40.5	7.79	52.0	19.5	375	18.6	34.7	607	5.9
5	F	2	20-Dec-17	9.6	8.7	0.3	0.6	91.4	1.6	7.0	152	40.7	7.88	51.6	19.4	375	18.6	34.2	701	5.9
6	F	2	20-Dec-17	6.9	5.7	0.2	1.0	83.4	2.1	14.5	143	37.7	7.72	48.9	18.6	380	18.0	31.0	656	5.4
7	F	3	20-Dec-17	6.9	5.5	0.2	1.2	79.2	2.4	18.4	154	40.9	8.30	49.2	18.6	378	19.2	32.7	737	5.7
8	F	3	20-Dec-17	7.1	5.5	0.3	1.3	76.9	4.8	18.3	141	36.9	7.28	50.7	19.4	383	18.5	33.6	543	5.7
9	F	3	20-Dec-17	7.2	5.8	0.3	1.1	81.3	2.5	16.2	145	38.8	7.60	51.1	19.1	374	18.7	33.8	697	5.7

The below parameters were analyzed and evaluated. Red cell distribution width (absolute) and Mean platelet volume were also analyzed as part of the test kit used for the analyses, but are reported in the Pathology evaluation.

Red blood cells	RBC
Red cell distribution width (%)	RDW%
Red cell distribution width (absolute)	RDWa
Mean red cell hemoglobin	MCH
Hematocrit	HCT
Mean red cell hemoglobin concentration	MCHC
Mean red cell volume	MCV
Hemoglobin	HGB
Platelets	PLT
Mean platelet volume	MPV
White blood cells	WBC
Lymphocytes	LYM
Granulocytes	GRAN
Monocytes	MONO

Appendix Table S3. Plasma chemistry data

SIG-41			Sample/	ALB	ALP	ALT	AMY	TBIL	BUN	CA	PHOS	CRE	GLU	NA+	K+	TP	GLOB
ID/Animal no	Gender	Group	Analysis dat	g/L	U/L	U/L	U/L	µmol/L	mmol/L	mmol/L	mmol/L	µmol/L	mmol/L	mmol/L	mmol/L	g/L	g/L
1	F	1	20-Dec-17	61	162	33	550	5	4,8	2,84	2,08	62	9,4	139	3,7	68	7
2	F	1	20-Dec-17	72	139	26	603	5	6,3	2,92	1,72	49	8,7	138	4,1	77	0
3	F	1	20-Dec-17	65	158	44	606	5	5,4	3,01	2,52	43	7,8	139	4,5	76	11
4	F	2	20-Dec-17	72	198	49	689	5	7,0	2,93	2,04	28	7,6	137	4,1	78	0
5	F	2	20-Dec-17	64	163	35	725	5	6,1	3,02	2,24	40	8,6	140	4,5	71	7
6	F	2	20-Dec-17	62	111	27	647	5	7,1	2,87	2,20	18	9,2	136	4,6	67	5
7	F	3	20-Dec-17	65	128	32	644	5	6,0	2,90	2,25	26	8,9	138	4,6	72	8
8	F	3	20-Dec-17	62	123	26	566	5	5,3	2,91	2,24	43	8,8	139	5,1	68	6
9	F	3	20-Dec-17	61	102	28	650	5	5,6	2,89	2,06	39	7,5	138	4,0	69	8

Albumin	ALB
Alanine aminotransferase	ALT
Alkaline phosphatase	ALP
Amylase	AMYL
Bilirubin (total)	TBIL
Blood urea nitrogen	BUN
Calcium	Ca^{++}
Creatinine	CREA
Globulin	GLOB
Glucose	GLU
Phosphate	PHOS
Potassium	K^{+}
Sodium	Na^{+}
Total protein	TP

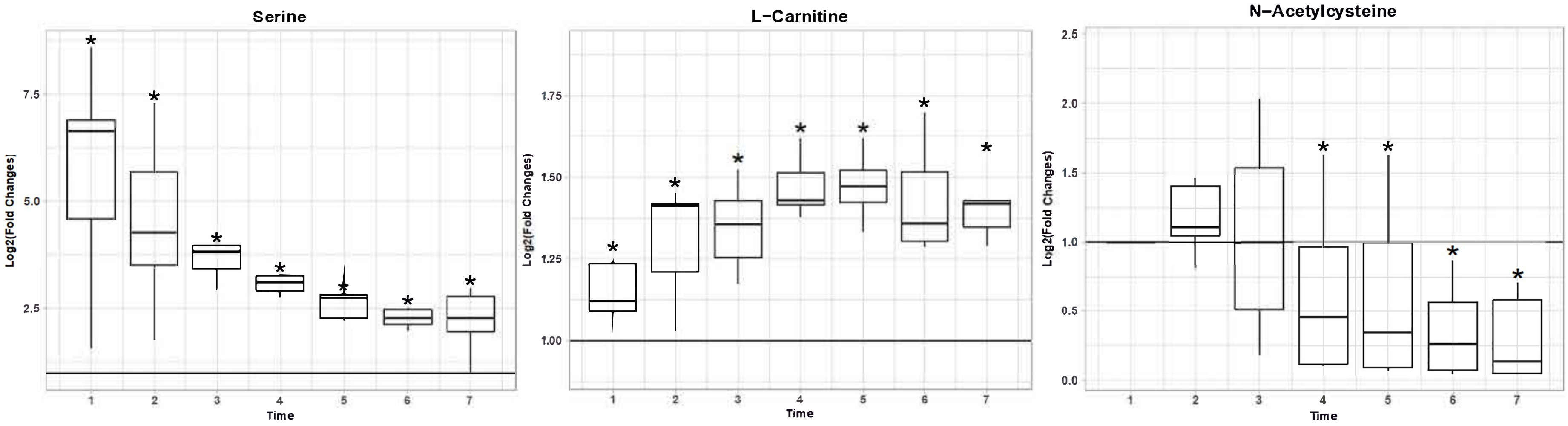

Appendix Figure S1. Changes in plasma level of each metabolic cofactor (except for NR) in supplementation study compared to baseline based on targeted metabolomics measurement.

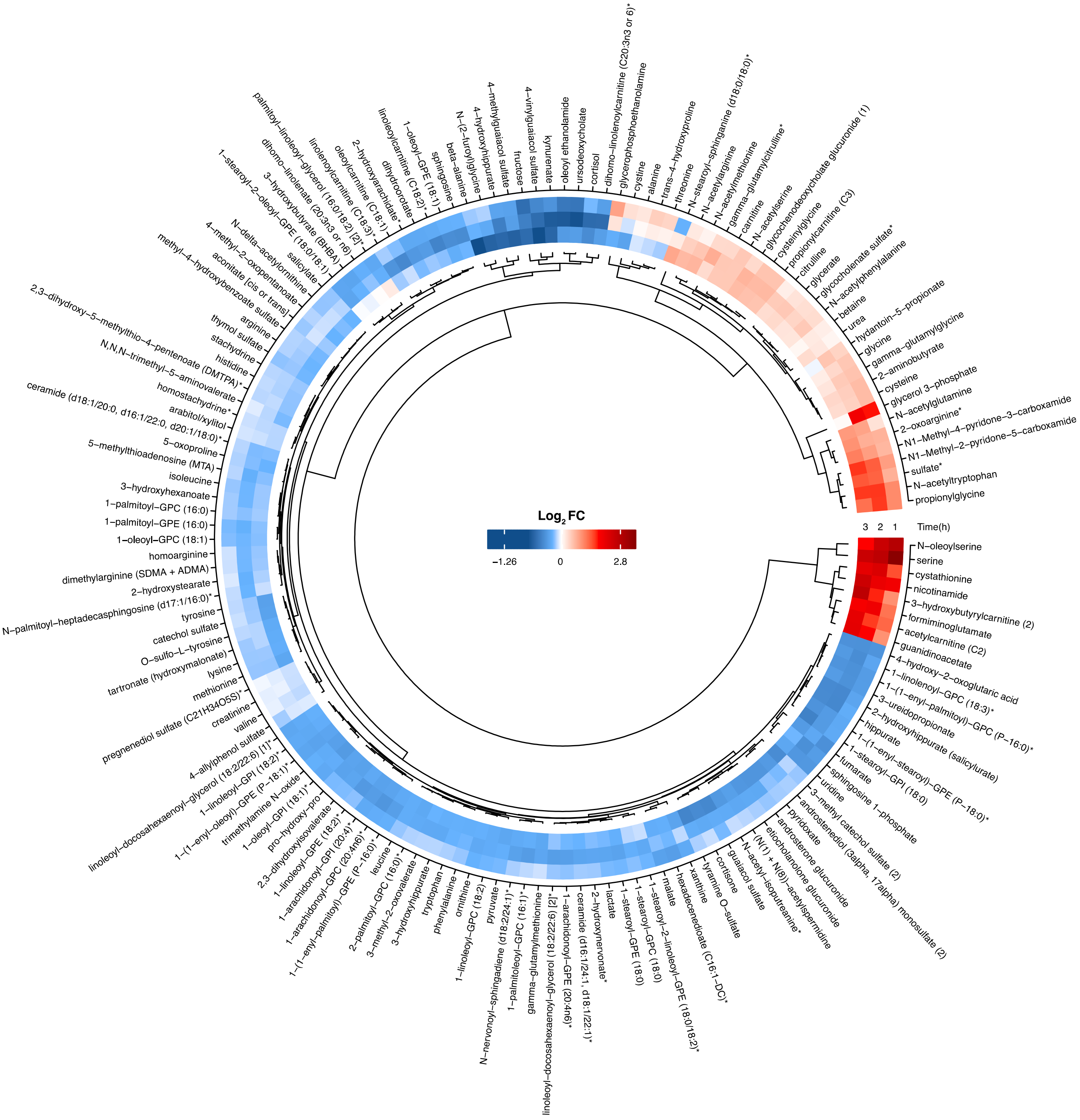

Appendix Figure S2. Changes in plasma level of metabolites in at least 2 out of 3 time points compared to baseline based on un-targeted metabolomics measurement. Identified metabolites were clustered based on hierarchical analysis using log2 FC as a distance metric. Significance level of P-value<0.05 after FDR adjustment.

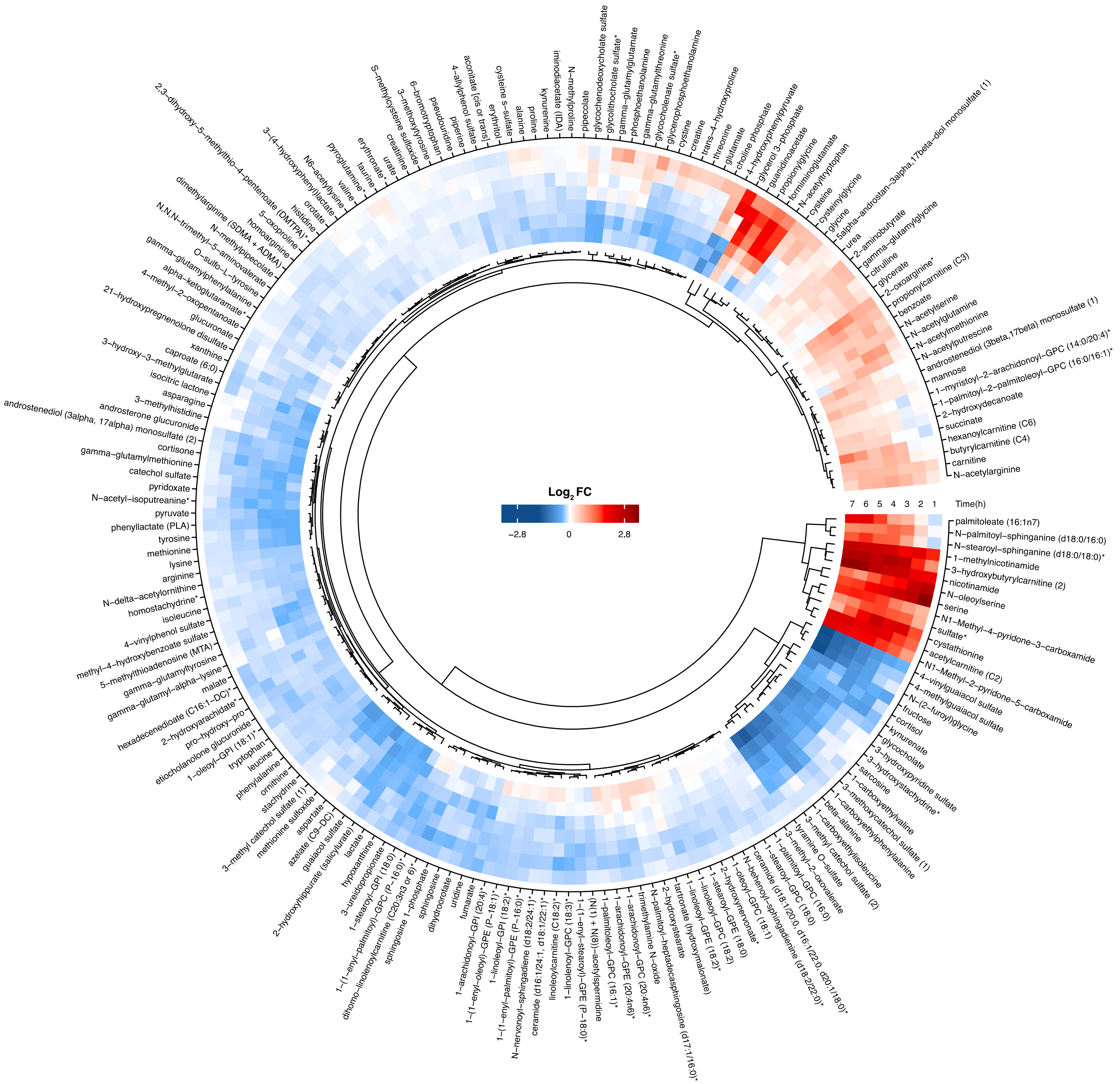

Appendix Figure S3. Changes in plasma level of metabolites in at least 3 out of 7 time points compared to baseline based on un-targeted metabolomics measurement. Identified metabolites were clustered based on hierarchical analysis using log2 FC as a distance metric. Significance level of P-value<0.05 after FDR adjustment.

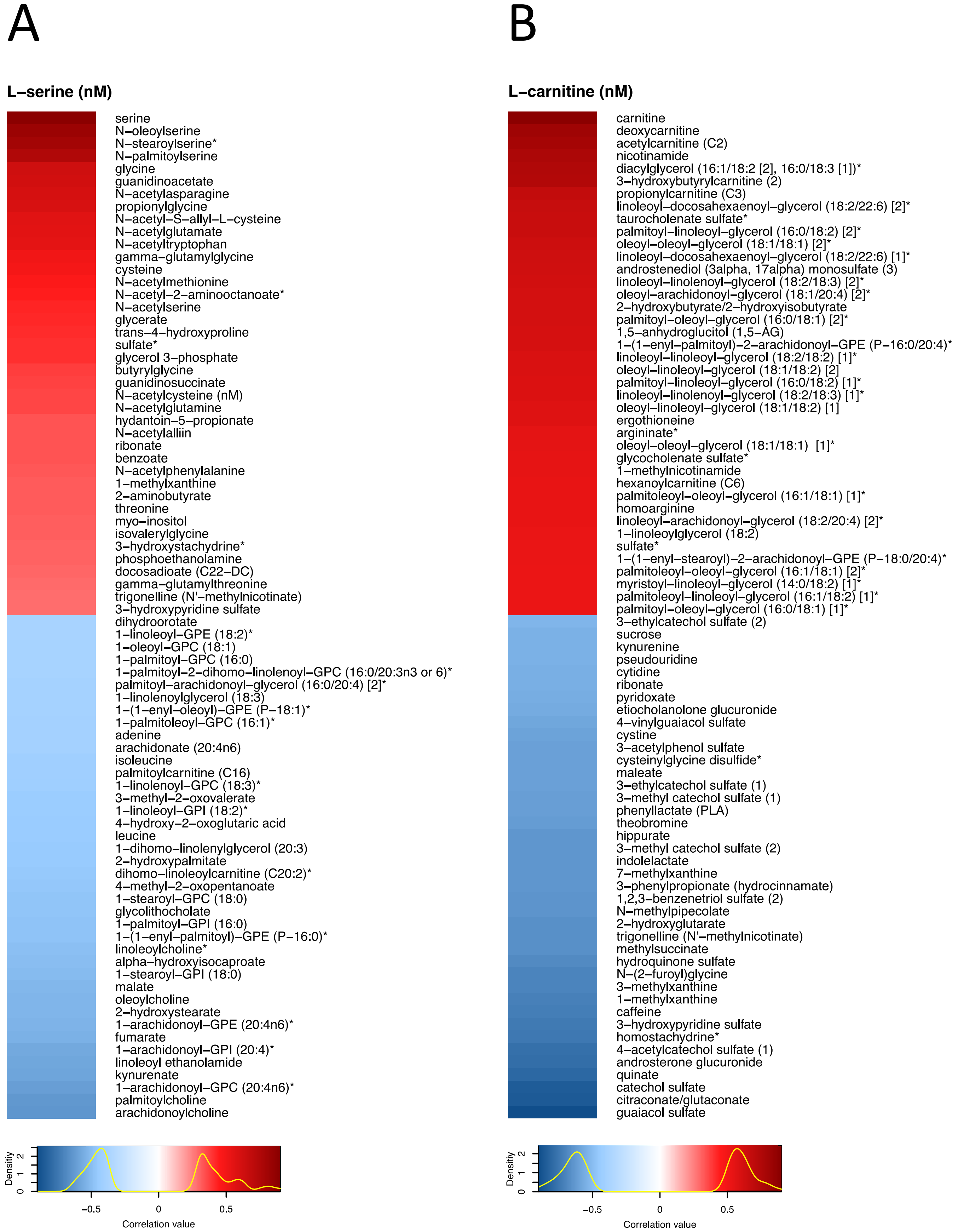

Appendix Figure S4. Top and bottom 40 metabolites (un-targeted & targeted metabolomics) that are significantly associated with supplemented L-Serine and L-Carnitine based on Spearman correlation analysis.

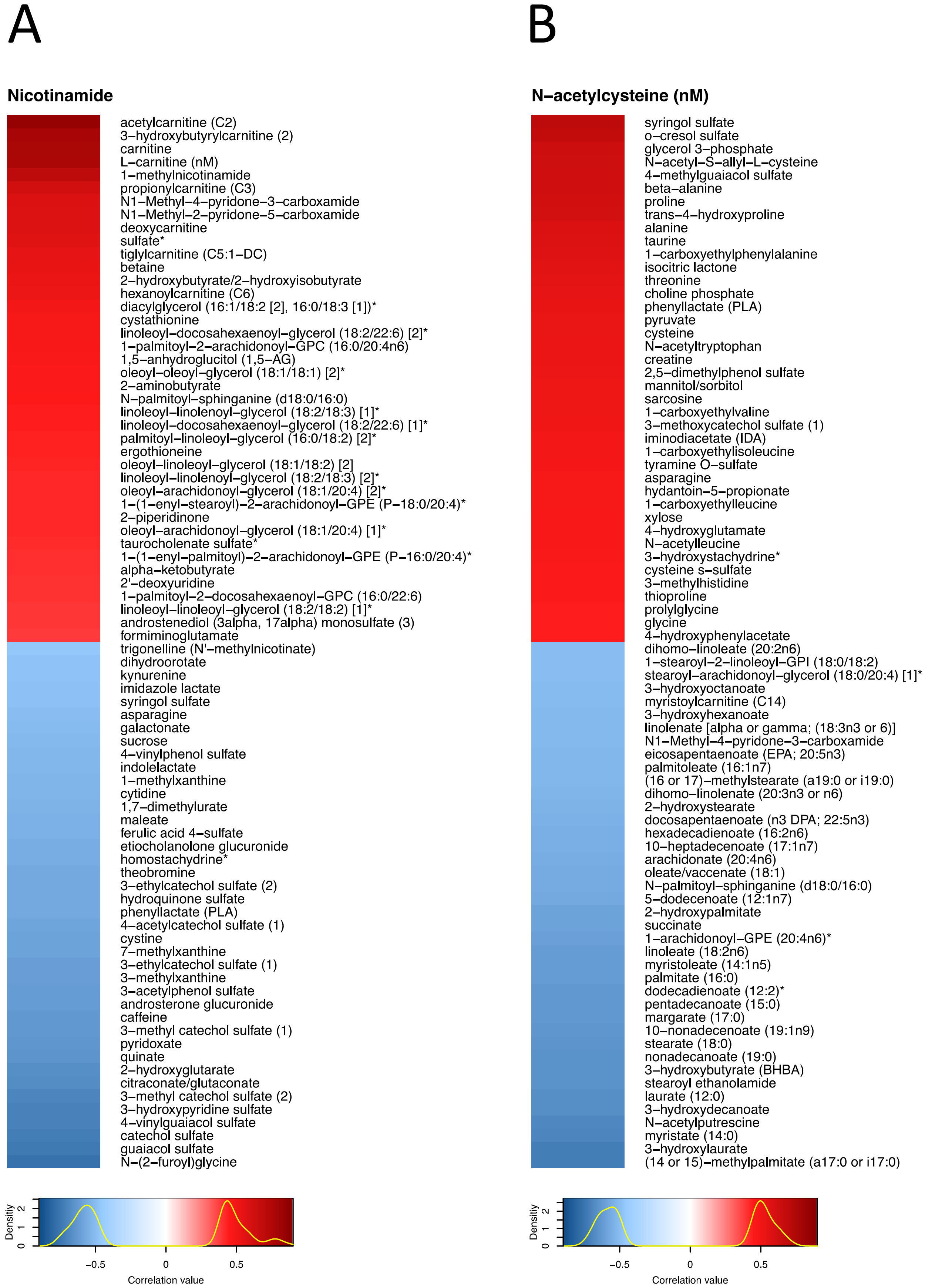

Appendix Figure S5. (A – B) Top and bottom 40 metabolites (un-targeted & targeted metabolomics) that are significantly associated with supplemented N-acetylcysteine and Nicotinamide based on Spearman correlation analysis.

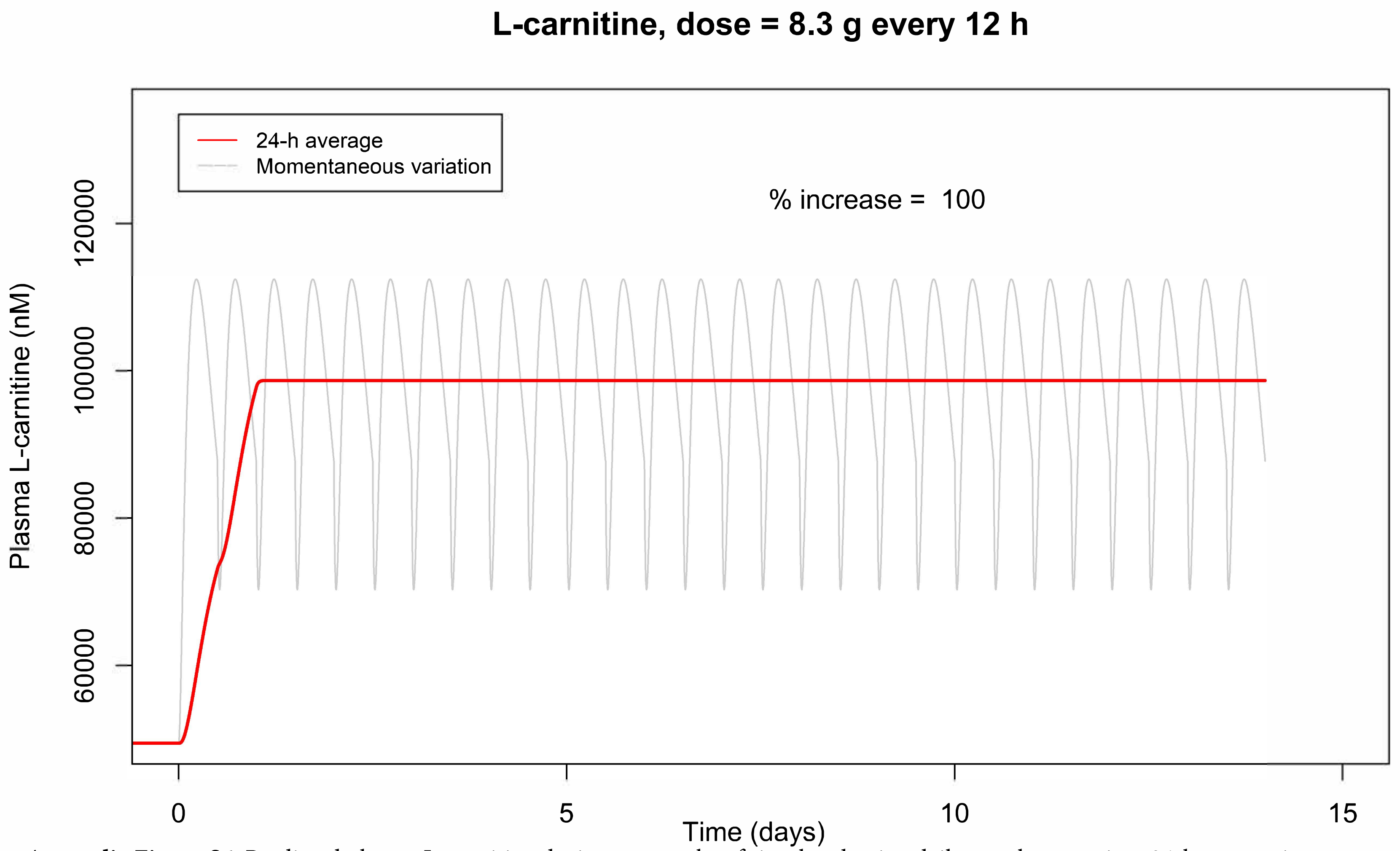

Appendix Figure S6. Predicted plasma L-carnitine during two weeks of simulated twice-daily supplementation: 24-hour moving average value indicated in red and continuous/momentaneous indicated in gray.
